# The TANGO-DM randomized controlled trial study protocol: treatment outcomes for gestational diabetes diagnosed according to WHO 2013 or WHO 1999 thresholds

**DOI:** 10.1186/s12884-025-07230-x

**Published:** 2025-02-17

**Authors:** Doortje Rademaker, Leon de Wit, Anne van der Wel, Eline van den Akker, Babette Braams-Lisman, Remke Dullemond, Inge Evers, Sander Galjaard, Brenda Hermsen, Marion van Hoorn, Anjoke Huisjes, Joepe Kaandorp, Annemiek Lub, Simone Lunshof, Flip van der Made, Remco Nijman, Judith van Laar, Karlijn Vollebregt, Joost Velzel, Floortje Vlemmix, Michelle Westerhuis, Lia Wijnberger, Maurice Wouters, Joost Zwart, Judith Bosmans, Patrick Bossuyt, Ruben Duijnhoven, Enrico Lopriore, Esteriek de Miranda, Corine Verhoeven, Ben Willem Mol, Arie Franx, J. Hans DeVries, Bas van Rijn, Rebecca Painter

**Affiliations:** 1 Department of Obstetrics and Gynecology, Amsterdam UMC location University of Amsterdam, Meibergdreef 9, Amsterdam, the Netherlands; 2 Amsterdam Reproduction and Development Research Institute, Amsterdam, the Netherlands; 3 Department of Obstetrics and Gynecology, WKZ, Lundlaan 6, Utrecht, 3584 EA the Netherlands; 4 Department of Obstetrics and Gynecology, OLVG, Amsterdam, the Netherlands; 5 Department of Obstetrics and Gynecology, Tergooi MC, location Hilversum, Hilversum, the Netherlands; 6 Department of Obstetrics and Gynecology, Jeroen Bosch Hospital, ’s-Hertogenbosch, the Netherlands; 7 Department of Obstetrics and Gynecology, Meander Medical Center, Amersfoort, the Netherlands; 8 Department of Obstetrics and Gynecology, Division of Obstetrics and Fetal Medicine, Erasmus MC University Medical Center, Rotterdam, the Netherlands; 9 Department of Obstetrics and Gynecology, Haga Hospital, The Hague, the Netherlands; 10 Department of Obstetrics and Gynecology, Gelre Hospitals, Apeldoorn, the Netherlands; 11 Department of Obstetrics and Gynecology, Flevoziekenhuis, Almere, the Netherlands; 12 Department of Obstetrics and Gynecology, Amphia hospital, Breda, the Netherlands; 13 Department of Obstetrics and Gynecology, Franciscus Gasthuis and Vlietland, Rotterdam, the Netherlands; 14 Department of Internal Medicine, Groene Hart Hospital, Gouda, the Netherlands; 15 Department of Obstetrics and Gynecology, Máxima Medical Center, Veldhoven, the Netherlands; 16 Department of Obstetrics and Gynecology, Spaarne Gasthuis, Haarlem, the Netherlands; 17 Department of Obstetrics and Gynecology, Noordwest hospital group, Alkmaar, the Netherlands; 18 Department of Obstetrics and Gynecology, Dijklander Hospital, Hoorn, the Netherlands; 19 Department of Obstetrics and Gynecology, Catharina Hospital, Eindhoven, the Netherlands; 20 Department of Obstetrics and Gynecology, Rijnstate Hospital, Arnhem, the Netherlands; 21 Department of Obstetrics and Gynecology, Amsterdam UMC location Vrije Universiteit, Boelelaan, Amsterdam, 1117 the Netherlands; 22 Department of Obstetrics and Gynecology, Deventer Hospital, Deventer, the Netherlands; 23 Department of Health Sciences, Faculty of Earth and Life Sciences, EMGO+ Institute for Health and Care Research, VU University Amsterdam, Amsterdam, the Netherlands; 24 Department of Epidemiology & Data Science, Amsterdam Public Health, Amsterdam University Medical Centres, University of Amsterdam, Amsterdam, the Netherlands; 25 Division of Neonatology, Leiden University Medical Centre, Leiden, The Netherlands; 26 Amsterdam UMC location Vrije Universiteit Amsterdam, Midwifery Science, De Boelelaan 1117, Amsterdam, the Netherlands; 27 Midwifery Academy Amsterdam Groningen, Inholland, Amsterdam, Netherlands; 28 Amsterdam Public Health research institute, Quality of Care, Amsterdam, Netherlands; 29 Department of Primary and Long Term Care, University of Groningen, University Medical Center Groningen, Groningen, Netherlands; 30 Division of Midwifery, School of Health Sciences, University of Nottingham, Nottingham, UK; 31 Department of Obstetrics and Gynecology, Monash University, Clayton, VIC Australia; 32 Aberdeen Centre for Women’s Health Research, Institute of Applied Health Sciences, School of Medicine, Medical Sciences, and Nutrition, University of Aberdeen, Aberdeen, Scotland; 33 Department of Internal Medicine, Amsterdam UMC location University of Amsterdam, Meibergdreef 9, Amsterdam, the Netherlands

**Keywords:** Gestational diabetes mellitus, WHO 1999, IADPSG, Randomized controlled trial, Large-for-gestational-age

## Abstract

**Introduction:**

Gestational diabetes mellitus (GDM), or hyperglycemia first diagnosed in pregnancy, affects 7–10% of all pregnancies worldwide. Perinatal risk rises with increasing glycemia at oral glucose tolerance test (OGTT). The new (2013) WHO criteria recommend a lower fasting, and a higher post-load threshold for GDM diagnosis in comparison to the old (1999) WHO criteria. To date, however, outcomes of GDM treatment for those affected by the altered diagnostic criteria, has not been well investigated. We hypothesized that intensive GDM treatment according to the new (2013) GDM criteria would result in a reduction in infants with birth weight > 90th centile (large for gestational age, LGA), in comparison to treatment according to the old criteria (1999).

**Methods:**

The TANGO-DM trial is an open label, multicenter randomized controlled trial. Participants are pregnant with a gestational age between 16 + 0 and 32 + 0 weeks, who underwent a 1-step venous 2- or 3-point 75-gram oral OGTT, were eligible if they had glucose concentrations discordant between the old (1999) and the new (2013) criteria. After informed consent, women are randomized to either intensive GDM treatment, consisting of dietary advice and glucose monitoring and, if euglycemia is not reached, antihyperglycemic agents, or normal obstetric care without GDM treatment. The primary outcome is large-for-gestational-age infants (birth weight > 90th percentile). Secondary outcome measures include maternal complications, obstetric complications, neonatal complications, obstetric interventions, quality of life, and healthcare and societal costs. Outcomes will be analyzed according to the intention-to-treat principle. The study is powered to detect a reduction in LGA from 16% in the untreated to 10% in the treated group, which requires 1032 participants (516 per arm; alpha-error 5% for 80% power).

**Discussion:**

The TANGO-DM trial will provide high-level evidence to support or refute the use of the new 2013 WHO diagnostic criteria in terms of their ability to lower the number of large for gestational age infants and/or improve maternal and perinatal outcomes and/or costs in women with gestational diabetes.

**Trial registration:**

Central Committee on Research Involving Human Subjects (CCMO) (NL63013.018.18). Registered on 22 September 2018.

**Supplementary Information:**

The online version contains supplementary material available at 10.1186/s12884-025-07230-x.

## Introduction

Gestational diabetes mellitus (GDM), or hyperglycemia first diagnosed in pregnancy, complicates approximately 7–10% of all pregnancies worldwide [1, 2]. GDM carries substantial maternal and perinatal risks, including large-for-gestational-age (LGA) neonates, shoulder dystocia, caesarean section, preeclampsia and neonatal hypoglycemia. Children born from pregnancies complicated by GDM have increased risks of obesity, diabetes and cardiovascular problems in later life [3, 4]. Treatment of GDM with a combination of dietary advice and, if necessary, glucose-lowering medication has been shown to decrease the incidence of some, but not all, adverse pregnancy outcomes, with some inconsistencies between studies [4, 5].

In 1999, the WHO released guidance for GDM diagnosis thresholds, which were informed primarily by the existing diagnostic guidelines for type 2 diabetes (DM2) [6, 7] In a large prospective international cohort, the Hyperglycemia and Adverse Pregnancy Outcomes (HAPO) study demonstrated a linear association between increasing maternal glucose concentrations at mid trimester oral glucose tolerance testing (OGTT) and increasing rates of LGA, neonatal hypoglycemia, caesarean section rate, and high cord serum C-peptide concentrations [8]. This association was also evident at glucose concentrations below the WHO 1999 threshold for GDM [8, 9]. Thus, in 2013, triggered by the HAPO findings, the WHO issued new diagnostic thresholds for GDM, based on the consensus that an increased odds ratio (1.75 compared to median glucose concentrations) for selected adverse pregnancy outcomes inferred that GDM treatment should be beneficial in an extended group [10]. Subsequently, most countries implemented these new diagnostic thresholds [1, 11].

Based on the 2013 WHO thresholds, an additional group of pregnant women with mild hyperglycemia, formerly labeled as normal glucose tolerance, are now classified as having GDM and will be advised treatment. As a result of adding the 1-hour value, there is a group of women who would not have been diagnosed because the 1-hour blood draw did not exist in the WHO 1999 guideline. In addition, there is a group of women formerly labeled as having GDM who are now classified as normal glucose tolerance. Hillier et al. showed that expanded criteria for GDM might almost double the incidence of GDM without lowering risk in adverse pregnancy outcome [12]. The recent GEMS trial showed similar results in a large group of women who were randomized with the use of lower or higher glycemic criteria for diagnosis. However, on closer examination of the GEMS subgroup analyses among participants with OGTT results discordant between the higher and lower criteria, allocation to intensive GDM treatment was found to lead to a large reduction in large for gestational age infants. The fact that the GEMS trial was not designed to investigate this discordant group hampers interpretation of this particular finding, which may be of relevance to individual patients [13]. Therefore, to date, there is no evidence from well-designed randomized trials, that treatment of GDM diagnosed by the new (2013) WHO criteria, but not by the old (1999) criteria, leads to improved perinatal and maternal outcomes. Thus, benefits and harms, and cost-effectiveness of adopting these new criteria are currently unknown [14–16].

The new (2013) WHO criteria have now been implemented in many, but not all, countries [17, 18]. The Netherlands, along with other European countries including the United Kingdom, Sweden, Denmark and Belgium, had, up until the start of this study (2018), not implemented the WHO 2013 criteria [19]. Since then, Sweden has implemented the WHO 2013 criteria, and Belgium has introduced a two-step method, in which at the second test the diagnosis is made with the WHO 2013 criteria.

There is thus an immediate and short-lived window of opportunity to assess the potential health benefits and economic costs of the WHO 2013 criteria.

## Methods

### Aims, design and setting

The TANGO-DM trial (randomized trial of effective treatment according to new GDM criteria) will evaluate the effectiveness of intensive GDM treatment as compared to normal obstetric care in pregnant women with a discordant OGTT result between the new (2013) WHO criteria as compared to the old (1999) criteria. This study will be conducted as an open label multicenter randomized controlled trial (RCT), applying an individual-randomization strategy. The study will be conducted within the framework Dutch Consortium for Healthcare Evaluation and Research in Obstetrics and Gynaecology – NVOG Consortium 2.0, a collaborative network of all major hospitals in the Netherlands and the Dutch Society of Obstetrics and Gynaecology (NVOG). The trial has been approved by the Medical Research Ethics Committee of the Amsterdam UMC, location AMC (METC 2018_173). The trial is registered at the Central Committee on Research Involving Human Subjects (CCMO) on 22 September 2018 under number NL63013.018.18 [20].

### Participants and eligibility criteria

**Fig. 1:**
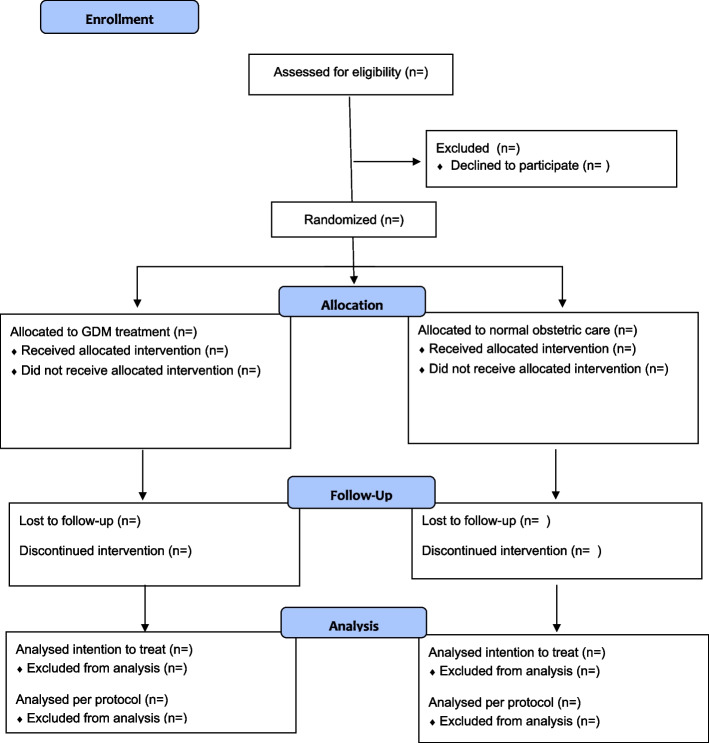
Consort flow diagram

A flowchart is shown in Fig. 1.

Women are eligible if they have a singleton pregnancy, with a gestational age between 16 + 0 and 32 + 0 weeks at study entry, aged *≥* 18 years, without pre-conceptional diabetes, a major fetal congenital abnormality, or significant medical or psychiatric co-morbidities as judged by the investigator (e.g., high dose corticosteroid treatment), with the ability to understand the informed consent form. In addition, participants will need to have undergone a 2- or 3-point 75-gram OGTT, consisting of a fasting, 1 h (in case of 3-point OGTT) and 2 h post load glucose concentration via venipuncture, conducted between gestational ages 16 + 0 and 30 + 0 weeks yielding glucose concentrations discordant between the old WHO (1999) and the new WHO (2013) criteria.

A discordant result is defined as any single blood glucose concentration during a 75-gram venous blood OGTT within the following ranges, in the absence of any other glucose concentration exceeding the upper limit of discordancy (Table 1). For example, a pregnant individual is eligible if she has a fasting glucose between 5.1 mmol/l and 7.0 mmol/l, as long as the 2-hour glucose is below 8.5 mmol/l. Similarly, a pregnant individual with a 2-hour glucose between 7.8 mmol/l and 8.5 mmol/l is eligible, as long as the fasting glucose is < 7.0 mmol/l.

**Table 1:** Glucose cut of values

	WHO 2013	WHO 1999
Fasting glucose	≥ 5.1 mmol/l	< 7.0 mmol/l
1-hour glucose	≥ 10 mmol/l	
2-hour glucose	< 8.5 mmol/l	≥ 7.8 mmol/l

As recommended by the Guideline ‘Diabetes in Pregnancy’ of the Dutch Society of Gynaecology and Obstetrics (NVOG) and the Royal Dutch Organisation of Midwives (Koninklijke Nederlandse Organisatie van Verloskundigen (KNOV)), obstetricians and midwives in the Netherlands screen for GDM between 24 and 28 weeks gestational age (or 16 weeks of gestation in case of history of GDM) using a risk-based, 1-step screening strategy [6]. Risk factors for GDM are defined in the guideline as: family history of diabetes mellitus in a first degree relative; history of GDM, history of LGA or macrosomia > 4.5 kg; obesity (body mass index (BMI) > 30 kg/m^2)^; polycystic ovarian syndrome (PCOS); ethnicity (established by self-identification) associated with high GDM prevalence (e.g. Afro-Caribbean, Hindu-Surinamese, South-Asian, Middle-Eastern, Moroccan, Egyptian) and a history of unexplained stillbirth. Furthermore, clinical symptoms (e.g. polyuria, polydipsia, suspected macrosomia and/or hydramnios) suggestive of diabetes, are to trigger an oral glucose tolerance test.

Although there is wide uptake of the risk factor-based screening strategy in the Netherlands, clinicians are at liberty to apply different screening strategies at their discretion or upon patient request, without affecting eligibility.

The study will be executed in healthcare settings with both mid/high risk pregnancies (hospital antenatal care) and low risk pregnancies (community midwife antenatal care).

### Recruitment and study procedure

**Fig. 2:**
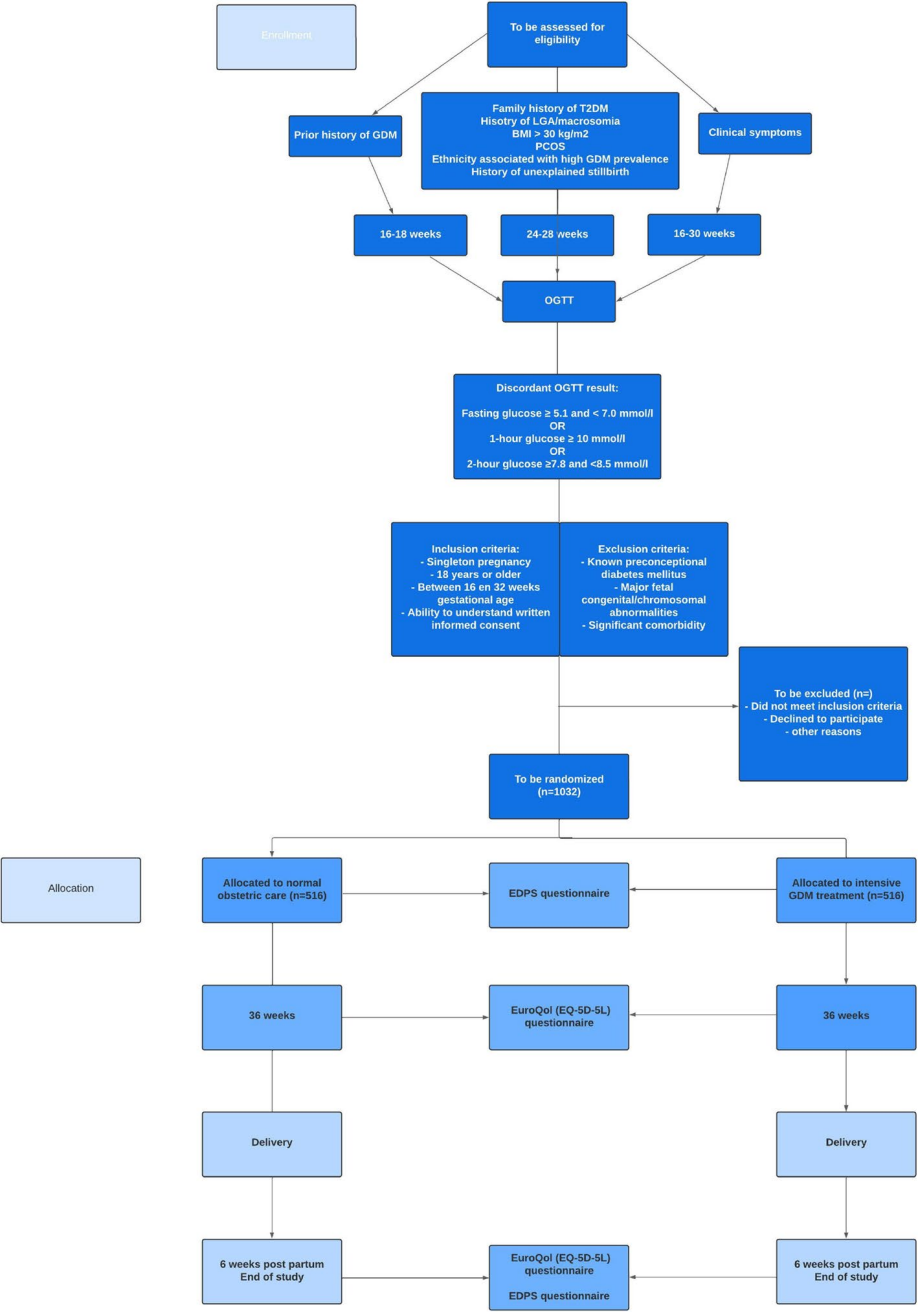
Flowchart screening and study procedures

A flowchart is shown in Fig. 2.

Eligible patients will be informed and invited to participate by either their diabetes care or obstetric care provider, i.e. physician, obstetrician, midwife, or diabetes nurse. Following counselling, written informed consent is obtained. Sufficient recruitment depends on the applicability of our assumptions of the number of pregnant women with discordant OGTT results. Based on work from the Netherlands, a conservative estimate of the percentage of women with a discordant blood glucose concentration after OGTT is around 9–15% [21].

Participants of the study will be randomized between:

1. Intensive GDM treatment
2. Normal obstetric care

#### Intensive GDM treatment

Participants randomized to intensive treatment will be counselled on the diagnosis of GDM and conventional GDM treatment will be initiated. Treatment will comply with the national guideline for GDM [22] and will include:

- Self-monitoring of blood glucose (target values: fasting *≤* 5.3, 1-hour postprandial *≤* 7.8 and/or *≤* 6.7mmol/l), or target values according to local protocol (e.g. 1.5-hour postprandial < 7 mmol/l). The recommended frequency for blood glucose measurements is (initially) daily, four times a day.
- Dietary recommendations [23].
- Pharmacotherapy for those unable to achieve euglycemia with dietary intervention alone: guidelines in the Netherlands suggest insulin as a first choice; oral antihyperglycemic agents are optional at the clinician’s discretion [6].

Mode and timing of delivery, postpartum advice, and postpartum screening for neonatal hypoglycaemia will be conducted based on the NVOG guideline for GDM and/or local protocol. Additional ultrasounds and induction of labor could be part of the intervention, but are not mandatory according to national guidance, which does recommend induction of labor before 39 weeks gestational age in case of GDM requiring medication. Postpartum recommendations after GDM include annual visits at the general practitioner to rule out diabetes type 2. Furthermore, in subsequent pregnancies after GDM, early OGTT screening is offered at 16 weeks, in addition to routine screening at 24–28 weeks.

In addition to the indications in standard care, postpartum screening for neonatal hypoglycaemia is recommended only for infants of GDM mothers treated with insulin or oral antihyperglycemic agents. Diet controlled GDM is not considered a routine indication for neonatal hypoglycemia screening [6].

#### Normal obstetric care

Participants randomized to normal obstetric care will receive maternity care by their usual care provider (midwife or obstetrician, as appropriate), according to common practice, local protocol and/or national guidelines [24, 25] for the duration of the pregnancy, birth and postpartum period. This includes that advice regarding future pregnancies, glucose testing and neonatal screening for hypoglycemia will only be offered in case of risk factors including macrosomia or small for gestational age. In contrast to intensive GDM care, in future pregnancies, early screening for GDM will not be advised, and yearly checkups at the general practitioner to rule out type 2 diabetes will not be indicated.

For participants randomized to normal obstetric care, who have been randomized early in pregnancy (16–20 weeks) and who have been tested because of a prior pregnancy complicated by GDM, a repeat OGTT will be carried out at 24–28 weeks of gestational age as part of routine antenatal care. Alternatively, a clinical indication may arise to repeat the OGTT later in pregnancy, for instance because of clinical symptoms. Among participants allocated to normal obstetric care, when glucose values are normal or discordant, normal obstetric care will be continued. Glucose values exceeding the discordant results (fasting *≥* 7.0 OR 2-hour post load glucose value *≥* 8.5 mmol/l) will result in initiation of GDM treatment (see Figs. 1 and 2), which will result in appropriate recommendations for future pregnancies, postnatal screening for diabetes type 2 and may include neonatal hypoglycemia screening.

#### Questionnaires

For all participants in the Tango-DM trial, questionnaires will be administered at 3 time points: study inclusion, around 36 weeks of pregnancy and 4–6 weeks postpartum.

Quality of life will be measured using the EuroQol (EQ-5D-5 L) at a gestational age of 36 weeks and 6 weeks post-partum. The EQ-5D-5 L assesses 5 domains (mobility, self-care, usual activities, pain/discomfort, anxiety/depression) with 5 possible answer levels ranging from “no problems” to “severe problems” [26].

Mood will be scored using the Edinburgh Postnatal Depression Scale (EPDS) at inclusion and post-partum. The EPDS has 7 questions regarding depression and anxiety [27].

The questionnaires will take approximately 10–30 min to complete.

#### Randomization

Randomization to the intervention arm or comparator arm will be done on a 1:1 basis, using the online software tool Castor EDC (www.castoredc.com, Castor EDC, Ciwit B.V., the Netherlands and Castor Research Inc, USA). Castor EDC complies with all applicable laws and regulations. Block stratified randomization will be used, with stratification for center and alternating variable block lengths of 2 and 4 with a block size unknown to the site. The trial is not blinded: health care providers and participants will be aware of their OGTT results and their allocation group.

We will register age, parity, prepregnancy BMI, prior GDM and ethnicity for women not consenting to randomization in the TANGO-DM trial.

### Outcome measures

#### Primary outcome measure

The primary outcome is large-for-gestational age birth weight (LGA, defined as birth weight > 90th centile, using the Dutch Perined reference charts, according to Hoftiezer [28]), retracted from the eCRF.

#### Secondary outcomes

Secondary outcomes will include all outcomes defined in the core outcome set for studies of gestational diabetes mellitus prevention and treatment; Table 2 [29].

**Table 2:** Secondary outcome

Gestational age at time of inclusion	In weeks and days
Adherence to the intervention	Did participants complete treatment according to allocation group until delivery: yes/no
Hypertensive disorders of pregnancy	Pregnancy induced hypertension, Pre-eclampsia (defined as by the ISSHP(30)), HELLP (Hemolysis, Elevated Liver enzymes, Low Platelets)
Requirement and type of pharmacological therapy for hyperglycaemia	If yes, oral medication, insulin, other
Gestational weight gain	In kilograms
Mode of birth	Spontaneous, assisted vaginal, caesarean (if instrumental: fetal distress or failure to progress)
Gestational age at birth	In weeks and days
Birth weight	In grams
Small for gestational age	Defined as birth weight < 10th centile, using the Dutch Perined reference charts, according to Hoftiezer(28)
Preterm birth	< 37 weeks (0 days) gestational age
Neonatal glucose postpartum	As a continuous outcome measure and as dichotomous outcome: moderate, glucose < 2.6 mmol/l; severe, glucose < 2.0 mmol/l(6)
Neonatal intravenous glucose	Yes/no
Stillbirth	Yes/no
Neonatal death	Yes/no
Secondary outcomes we will report in addition to the core outcome set:	
Macrosomia	Birth weight > 4500 g
Reason for preterm birth	Medically indicated or spontaneous contractions and/or spontaneous rupture of membranes
Onset of labour	Spontaneous, planned Caesarean section, induction of labour
Method(s) of induction of labour	Foley catheter/mechanical, prostaglandines, amniotomy, oxytocin, other (more than one optional)
Postpartum hemorrhage	> 1000 ml blood loss
Puerperal sepsis requiring antibiotics	Yes/no
Maternal admission postpartum	Yes/no, if yes: duration in days
1-min Apgar score, 5-min Apgar score, 10-min Apgar score	
Shoulder dystocia	Yes/no
Birth injury	i.e. fractures, nerve palsy
Cord blood C peptide	In a subset, where available
Neonatal intensive care/medium care admission	Yes/no, if yes: duration in days.
Hyperbilirubinemia	Yes/no
Respiratory support > 24 h	Yes/no
Neonatal encephalopathy	Yes/no
Other:	serious health outcomes up to the time of primary hospital discharge assessed from patient medical records(31)

#### Baseline characteristics

Maternal baseline characteristics will be collected from the electronic patient file. We will provide a tabular presentation of the following baseline characteristics of both allocation groups, as well as the outcome variables: prepregnancy BMI (in kg/m^2^) and BMI at study entry, maternal age, parity, systolic and diastolic blood pressure at baseline and at study entry, intoxications (smoking, alcohol use), risk factors for GDM, reason for GDM screening, history of psychological problems, conception: spontaneous, fertility treatment (clomifene citrate, gonadotropins, IVF, ICSI), blood glucose concentrations at OGTT, gestational age at time of OGTT.

### Follow-up

To allow for long-term follow-up of both mothers and infants of the trial, all patients are asked for written consent to be approached for future studies with this purpose. However, follow up is not part of the current study, which ends at 6 weeks postpartum.

### Cost-effectiveness

The aim of the economic evaluation is to assess the incremental costs and the incremental health effects of intensive GDM treatment as compared to normal obstetric care in participants. Both a cost-effectiveness analysis (CEA) and a cost-utility analysis (CUA) will be performed from a societal and healthcare perspective according to Dutch guidelines [30] from diagnosis of GDM until 6 weeks postpartum. Because the time horizon of the economic evaluation is less than 12 months, discounting is not necessary.

Costs will be measured from a societal perspective using (web-based) questionnaires adapted from the iMTA Medical and Productivity Cost Questionnaires at a gestational age of 36 weeks and 6 weeks postpartum [26, 31]. Cost categories that will be included are: (1) healthcare costs (primary and secondary care, complementary cares and home care) (2) lost productivity costs (absenteeism from paid and unpaid work, and presentism) and (3) patient costs (informal care and other care services paid for by patients themselves). Valuation will be done according to Dutch costing guidelines [32]. For the valuation of health care utilization, lost productivity and informal care, Dutch standard costs will be used. Medication use will be valued using prices of the Royal Dutch Society for Pharmacy. Patient and family costs other than informal care will be valued using self-reported prices. For the valuation of absenteeism from paid work, the friction cost approach will be used.

The following effect measures will be included in the economic evaluation:

1. LGA (birth weight > 90th percentile)
2. Perinatal and maternal outcomes as stated in the section above
3. Quality-Adjusted Life-Years (EuroQol (EQ-5D-5 L) with Dutch reference values) [31]

Missing cost and effect data will be imputed using multiple imputation according to the MICE algorithm developed by van Buuren [33]. Rubin’s rules will be used to pool the results from the different multiply imputed datasets. Bivariate regression analyses will be used to estimate cost and effect differences between intensive GDM treatment and normal obstetric care while adjusting for confounders if necessary. Incremental cost-effectiveness ratios (ICERs) will be calculated by dividing the difference in the mean total costs between the treatment groups by the difference in mean effect between the treatment groups. Bias-corrected and accelerated bootstrapping with 5000 replications will be used to estimate 95% confidence intervals around the cost differences and statistical uncertainty surrounding the ICERs. Uncertainty surrounding ICERs will be graphically presented on cost-effectiveness planes. Cost-effectiveness acceptability curves will also be estimated showing the probability that, in pregnant women with discordant OGTT results, intensive GDM treatment is cost-effective in comparison to normal obstetric care for a range of different ceiling ratios thereby showing decision uncertainty [34].

### Burden of treatment

We anticipate that the treated group will perceive their pregnancies as more intrusive and potentially harbor negative sentiments towards their pregnancies in contrast to women receiving standard obstetric care. With the Quality-Adjusted Life-Years (EuroQol (EQ-5D-5 L) with Dutch reference values) and the Social support questionnaire (SSQ, send 6 weeks post-partum) we will evaluate the differences between the treated and untreated group.

### Safety and monitoring

To safeguard the interests of the trial participants, a data safety monitoring board (DSMB) monitors the progress of the trial’s recruitment, and periodically assesses adverse events in order to safeguard against any treatment harm during the trial. A safety review is performed by an independent statistician and reviewed by the DSMB after the results are available for 50% of the patients. No interim analysis for efficacy is planned. This sample size was calculated to provide power to detect differences in a surrogate outcome, LGA, as opposed to safety outcomes. In the case of a statistically significant difference in LGA at an interim analysis, this will not be considered a reason to stop the study.

All serious adverse events (SAE) reported by participants or observed by the investigators or staff will be recorded. SAE definitions and standards for expedited reporting follow the international conference of harmonization good clinical practice (ICH GCP) guidelines on safety reporting [35]. All SAEs are reported to the accredited ethics committee that approved the protocol, according to the requirements of that committee.

### Sample size

In the planning phase of the study, we initially calculated a sample size of 2,520 participants (1,260 per group). This would generate sufficient power to detect a reduction of the primary outcome LGA from 14% in the group randomized to normal obstetric care to 9.8% in the group randomized to intensive GDM treatment among participants (alpha = 0.05, beta = 0.2). The assumed prevalence of LGA in the group randomized to normal obstetric care was based on the RESPECT study [36], in which a 5% prevalence of GDM was found in a cohort of patients from midwifery practices and secondary and tertiary hospitals in the Utrecht region of the Netherlands, according to the old (1999) criteria. The prevalence of LGA among women with GDM in the RESPECT cohort was 19%. Reasoning that the prevalence of LGA in women with a discordant OGTT results is likely to be lower than the mean for GDM patients, but higher than the general population (e.g. 10%) led us to an estimate of 14% in women without treatment. This estimate is confirmed by unpublished analyses within the RESPECT cohort, which found a 16% prevalence of LGA among discordant cases.

However, the subsequently published GEMS trial [13], which contained data on the LGA prevalence in discordant cases, triggered our power recalculation. The population in the sub-analyses of the GEMS trial with discordant test results was more similar to the population in the TANGO-DM than the previously used numbers of the RESPECT cohort. The prevalence of LGA in women with untreated mild hyperglycemia in the GEMS trial was 18.0% compared to 6.2% in the treated group. We therefore used a background incidence of 16% LGA (instead of the earlier 14%) in untreated women, to demonstrate or refute a reduction of LGA to 10% in participants who are treated and a 5% correction for women who are lost to follow up, we need 1032 participants (516 per group). The recalculation of the sample size was not informed by interim data of the TANGO-DM trial. The adjustment was approved by the Medical Research Ethics Committee of the Amsterdam UMC, and changed in the trial registration.

### Analyses and reporting of results

#### General outline of analysis

The intention-To-Treat (ITT) population will consist of all eligible participants who gave consent and have been allocated to one of the two treatments, irrespective of treatment received. The per protocol population is defined as all participant who received the allocated treatment. The per protocol population includes those who initiated treatment for GDM as allocated following randomization and who completed the treatment until delivery, as well as those receiving normal antenatal care after having been allocated to the normal antenatal care group. If, in the course of normal antenatal care, a repeat OGTT was deemed necessary, and this repeat OGTT indicated a diagnosis according to both old and new GDM criteria, GDM treatment should be initiated as a part of normal antenatal care, and would not be a reason for a participant to be excluded from the per protocol analyses.

Participants who did not initiate GDM treatment when they had been allocated to GDM treatment, will be removed from the per-protocol population.

#### Planned analyses

Baseline characteristics will be presented as frequencies and percentages in each group, or as averages (mean or median) with standard deviations or interquartile ranges as appropriate. The primary outcome will be evaluated by estimating the relative risk between both treatment arms. Analyses will be conducted in both the intention-to-treat and per protocol population for the primary outcome.

Relative risks will be estimated for categorical outcomes, with 95% confidence intervals, and χ2 tests for significance. Fisher’s exact test will be used for sparse events where assumptions of the χ2 test are not met. Continuous data will be described as means with standard deviation if normally distributed, or medians with interquartile ranges when not normally distributed; tests for significance are t-tests or Mann-Whitney U tests, respectively. The Hodges-Lehman estimator will be used to calculate the confidence interval for the difference in medians.

Individuals who were randomized to normal obstetric care but for unknown reasons received treatment they will be excluded from the per protocol analysis. If the treatment followed an abnormal repeat OGTT they will remain in the per protocol analysis. Individuals who were randomized to GDM treatment but for unknown reasons never received treatment will be excluded from the per protocol analysis. If, per example, they did not receive treatment because of preterm birth they will remain in the per protocol analysis.

Subgroup analyses will be performed for BMI (under or above 30 kg/m2), early or late GDM (before or after 24 weeks) and according to the result of the OGTT (abnormal fasting result, abnormal postprandial result, or both).

#### Reporting of results

Reporting of results will be according to the CONSORT 2010 checklist of information to include when reporting a randomized trial.

### Data handling

A web-based electronic case record form (eCRF), Castor EDC, will be used to record baseline data including participants’ demographics, obstetrics and medical history, details regarding the pregnancy, and birth outcomes. The full eCRF is provided as a supplemental file (Supplement file 1). Allocation of unique participant identification numbers ensures participant’s privacy. The participant identification numbers are used in all study documentation. This code is only available to the local investigator and research staff. Periodic visits of an independent external study monitor to participating centres enables assessment of data quality and auditing of trial procedures.

### Ethics and dissemination

The Medical Research Ethics Committee (MREC) of the Amsterdam UMC, location AMC approved this trial. Trial reference number: METC 2018_173. Changes to the study protocol are documented in amendments. Amendments are submitted for approval to the MREC. Major changes will be updated on the trial registration website [37]. The full study protocol, including amendments, is publically available on the study website [38].

After completion of the trial the core investigators will report on the results of the main study and submit a manuscript to a peer-reviewed medical journal. Supplementary analyses will be reported separately.

## Discussion

The TANGO-DM trial is the first to provide randomized evidence on the effectiveness and cost-effectiveness of intensive GDM treatment versus no treatment in a group of women that would formerly i.e. before the new (2013) WHO criteria would not have been diagnosed as GDM, and subsequently would not have received treatment. It will provide guidance for the justification of these new diagnostic criteria and the study results will have important clinical implications. Moreover, the cohort formed by participants of the TANGO-DM trial provides an opportunity to assess differences in long-term health outcomes mothers and their offspring after treatment of no treatment.

Strengths of this trial include the large sample size and randomized design. We will report on all core outcome measures defined for GDM studies and, next to the effectiveness manuscript, provide a cost-effectiveness analysis [29]. The study did not proceed as originally planned due to the impact of the complexities associated with managing a large, multidisciplinary trial. The study involved multiple disciplines, including gynecology, midwifery, internal medicine, and clinical chemistry, which added complexity to its execution.

A limitation of this study may be non-compliance with voluntary questionnaires sent during pregnancy and the postpartum period. Compliance to fill in the questionnaires will be enhanced by sending questionnaires per email that can be filled in online and are compatible with smartphone.

GDM presents an ever-growing global problem, complicating up to 7–10% of pregnancies, and affecting 21 million pregnancies around the globe each year [39]. We expect that, in the Netherlands, the annual number of affected pregnancies, after replacing the old (1999) by the new (2013) WHO criteria for GDM, could expand by 3400 to 8500 cases. We assume that the prevalence of the primary outcome LGA is 16% in the group that receives standard obstetric care as compared to 10% in the group that receives intensive GDM treatment among women with a discordant GDM diagnosis between the old (1999) and new (2013) WHO criteria. Furthermore, we assume that the GDM treatment cost amount to an additional annual investment of 7 million euros in the Netherlands alone. What the TANGO-DM study will add to existing evidence, is whether GDM treatment, among those diagnosed according to the new WHO criteria, offers advantages in terms of limiting LGA in whether these benefits are cost-effective, for example by limiting health care expenditure for maternal, fetal and neonatal complications. Our trial findings are imperative to patients, clinicians and health care systems in shaping national and international GDM management policy decisions.

## Supplementary Information

Supplementary Material 1.

## Data Availability

TANGO-DM has a data sharing policy. At the end of the trial, the original locked database will be retained at Amsterdam University Centre. The datasets used and/or analyzed during the current study available from the corresponding author on reasonable request. All inquiries should be addressed to the principal investigator, Prof. R.C. Painter.
