# Supplementary material for: The TANGO-DM randomized controlled trial study protocol: treatment outcomes for gestational diabetes diagnosed according to WHO 2013 or WHO 1999 thresholds
Source: BMC Pregnancy Childbirth. 2025 Feb 17;25:173. doi: 10.1186/s12884-025-07230-x (PMC11834261; doi:10.1186/s12884-025-07230-x)
Supplement: Supplementary file 1 — Supplementary Material 1. [file 12884_2025_7230_MOESM1_ESM.pdf]

Tango-DM_dev - version 100.51

Printed on 25-09-2018 14:49:57 by Doortje Rademaker

1. TANGO-DM - General information

Number	Question	Answers
1.1	Age at time of randomization<br>*Warning shown if field's value is smaller than 18: 'The patient can not be included in the trial if below 18'*<br>*Notice shown if field's value is larger than 45: 'Age 45 years is outside the expected range, please check the age'*	
1.2	Estimated date of delivery (uitgerekende datum)<br>*Warning shown if field's value is smaller than or equal to 2017-01-01: 'Estimated date of delivery entered does not correspond with the study period. Please check the estimated date of delivery.'*<br>*Warning shown if field's value is larger than 2023-01-01: 'Estimated date of delivery entered does not correspond with the study period. Please check the estimated date of delivery.'*	*(dd-mm-yyyy)*
1.3	Please register the e-mailadress of the participant<br>*Warning shown if field's value is not equal to done: 'The email is needed to be able to send questionnaires, please contact the patient to add it'*	☐ done
	Fill in OGTT results to check inclusion criteria	
1.4	Fasting glucose<br>*Warning shown if field's value is smaller than 1: 'A value between 1 and 25 is expected, please correct'*<br>*Warning shown if field's value is larger than 25: 'A value between 1 and 25 is expected, please correct'*	mmol/l
1.5	1-hour glucose<br>*Warning shown if field's value is smaller than 1: 'A value between 1 and 25 is expected, please correct'*<br>*Warning shown if field's value is larger than 25: 'A value between 1 and 25 is expected, please correct'*	mmol/l
1.6	2-hour glucose<br>*Warning shown if field's value is smaller than 1: 'A value between 1 and 25 is expected, please correct'*<br>*Warning shown if field's value is larger than 25: 'A value between 1 and 25 is expected, please correct'*	mmol/l
1.7	Discordant result:	
	Patient is discordant	

1.7.2	***If 'Discordant result:' is equal to '0' answer this question:***<br>Discordant result:<br>*Warning shown if field's value is equal to 3: 'Patient is not discordant and can not be included'*<br>*Warning shown if field's value is equal to 0: 'One of the values is out of range, the patient can not be included'*	
1.7.3	***If 'Discordant result:' is equal to '3' answer this question:***<br>Discordant result:<br>*Warning shown if field's value is equal to 3: 'Patient is not discordant and can not be included'*<br>*Warning shown if field's value is equal to 0: 'One of the values is out of range, the patient can not be included'*	
1.7.4	***If 'Discordant result:' is equal to '2' answer this question:***<br>Discordant result:<br>*Warning shown if field's value is equal to 2: 'The values indicate patient has gestational diabetes and can not be included'*	

2. TANGO-DM - In-exclusion

Number	Question	Answers
	A patient must meet all the following criteria in order to be eligible for study inclusion	
2.1	Age 18 years or older<br>*Exclude patient if field's value is equal to no with message: 'The patient is not eligible for study inclusion'*	○ yes<br>○ no
2.2	Singleton pregnancy<br>*Exclude patient if field's value is equal to no with message: 'The patient is not eligible for study inclusion'*	○ yes<br>○ no
2.3	Date OGTT<br>*Warning shown if field's value is larger than NOW: 'The date is expected to be in the past, please correct'*	[ ] [ ] [ ] *(dd-mm-yyyy)*
2.4	Gestation age between 16 and 32 weeks<br>*Exclude patient if field's value is equal to no with message: 'The patient is not eligible for study inclusion'*	○ yes<br>○ no
2.5	Ability to understand written informed consent<br>*Exclude patient if field's value is equal to no with message: 'The patient is not eligible for study inclusion'*	○ yes<br>○ no
	If a patient meets any of the exclusion criteria, she is not eligible for study inclusion	
2.6	Known pre-existent diabetes mellitus<br>*Exclude patient if field's value is equal to yes with message: 'The patient should be excluded'*	○ yes<br>○ no

2.7	Severe medical or psychological comorbidity<br>*Exclude patient if field's value is equal to yes with message: 'The patient should be excluded'*	○ yes<br>○ no
2.8	Fetus with major congenital birth defect and/or chromosomal abnormalities<br>*Exclude patient if field's value is equal to yes with message: 'The patient should be excluded'*	○ yes<br>○ no
	Informed consent	
2.9	Patient has provided written informed consent<br>*Exclude patient if field's value is equal to no with message: 'The patient is not eligible for study inclusion'*	○ yes<br>○ no
2.9.1	***If 'Patient has provided written informed consent' is equal to 'yes' answer this question:***<br>Date of informed consent<br>*Warning shown if field's value is smaller than 2017-01-01: 'The date should be after the study-start 01-01-2017'*<br>*Warning shown if field's value is larger than 2023-01-01: 'The date should be before the study-enddate 01-01-2023'*<br>*Warning shown if field's value is larger than NOW: 'The date should not be in the future, please correct'*	☐ ☐ ☐ *(dd-mm-yyyy)*
2.10	Check of the criteria	

3. TANGO-DM - Randomization

Number	Question	Answers
3.1	Is randomization allowed	
	This page will only show details if all criteria are ok	
3.1.2	***If 'Is randomization allowed' is equal to 'yes' answer this question:***<br>Confirm you are ready to randomize the patient	○ Ready for randomization
	Please click on tab on the top right to perform the randomization	
3.1.2.2	***If 'Confirm you are ready to randomize the patient' is equal to 'Ready for randomization' answer this question:***<br>Randomized treatment	

3.1.2.3	***If 'Confirm you are ready to randomize the patient' is equal to 'Ready for randomization' answer this question:***<br>Randomization completed<br>*Warning shown if field's value is equal to no: 'Please check previous form on how to trigger randomization. All inclusion and exclusion criteria should be met and also Informed consent given.'*	○ yes<br>○ no
3.1.2.3.1	***If 'Randomization completed' is equal to 'yes' answer this question:***<br>Randomization date<br>*Warning shown if field's value is larger than 2023-01-01: 'The date should be before 1 January 2023'*<br>*Warning shown if field's value is smaller than 2017-01-01: 'The date should be after 1 January 2017'*	☐ ☐ ☐ *(dd-mm-yyyy)*
3.1.2.3.1.1	***If 'Randomization date' is not equal to '""' answer this question:***<br>Is Randomization date before date Informed Consent check<br>*Warning shown if field's value is equal to yes: 'The randomization date can not be before the date of signing Informed Consent. Please check date of informed consent and date of randomization.'*	
3.1.2.3.1.1.1	***If 'Is Randomization date before date Informed Consent check' is equal to 'yes' answer this question:***<br>Is Randomization date before date Informed Consent<br>*Warning shown if field's value is equal to yes: 'The randomization date can not be before the date of signing Informed Consent. Please check date of informed consent and date of randomization.'*	

4. TANGO-DM - Indication

Number	Question	Answers
	This page will only show details if all criteria are ok	
4.1	***If 'Is randomization allowed' is equal to 'yes' answer this question:***<br>Weight at time of study inclusion<br>*Warning shown if field's value is smaller than 40: 'A weight between 40 and 200 is expected'*<br>*Warning shown if field's value is larger than 200: 'A weight between 40 and 200 is expected'*	☐ kg

4.2	***If 'Is randomization allowed' is equal to 'yes' answer this question:***<br>What was the main reason to perform an OGTT? (multiple selections are allowed)	☐ suspected macrosomia/estimated fetal weight >p90 (current pregnancy)<br>☐ family history with diabetes<br>☐ obesity<br>☐ polyhydramnion<br>☐ prior pregnancy with GDM<br>☐ history of LGA or macrosomia<br>☐ polycystic ovarian syndrome<br>☐ history of unexplained stillbirth<br>☐ presence of other clinical symptoms (e.g. polyuria, polydipsia, suspected macrosomia)<br>☐ ethnicity<br>☐ other
4.3	***If 'Is randomization allowed' is equal to 'yes' answer this question:***<br>Ethnicity: please use other if country of birth is known and the ethnicity does not fit in the other categories	○ Caucasian<br>○ Indian/Pakistani/Bangladesi<br>○ Afro-Caribbean (Antilles, Surinam-creole)<br>○ Hindu/Caribbean (Surinam Hindu)<br>○ African (Sub-Sahara)<br>○ Middle Eastern/North African (Turkish, Moroccan)<br>○ Asian<br>○ other

5. TANGO-DM - Medical history

Number	Question	Answers
	This page will only show details if all criteria are ok	
5.1	***If 'Is randomization allowed' is equal to 'yes' answer this question:***<br>Diagnosis of Polycystic Ovary Syndrome (PCOS)	○ yes<br>○ no<br>○ unknown
5.2	***If 'Is randomization allowed' is equal to 'yes' answer this question:***<br>Thyroid problems: hypo- or hyperthyroidism	○ Hypothyroidism<br>○ Hyperthyroidism<br>○ thyroid problem but type is unknown<br>○ no<br>○ unknown

5.3	***If 'Is randomization allowed' is equal to 'yes' answer this question:***<br>History of psychological problems	☐ depression<br>☐ anxiety disorder<br>☐ other<br>☐ none<br>☐ unknown
5.4	History of psychological problems consistent	
5.4.1	***If 'History of psychological problems consistent' is equal to 'no' answer this question:***<br>History of psychological problems consistent<br>*Warning shown if field's value is equal to no: 'None should not be combined with other options, please correct'*	
5.5	***If 'Is randomization allowed' is equal to 'yes' answer this question:***<br>Chronic or pre-existent hypertension	○ yes (requiring medication)<br>○ yes (not requiring medication)<br>○ no<br>○ unknown
5.6	***If 'Is randomization allowed' is equal to 'yes' answer this question:***<br>Maternal medication use (other than folic acid and vitamins) during pregnancy	☐ no<br>☐ aspirin (acetylsalicylzuur)<br>☐ levothyroxine / thyrax<br>☐ SSRI (including sertraline, (es)citalopram, paroxetine, fluoxetine)<br>☐ Tricyclic antidepressant (including amitryptiline, nortriptyline)<br>☐ other<br>☐ unknown
5.7	MaternalMH_medication consistent	
5.7.1	***If 'MaternalMH_medication consistent' is equal to 'no' answer this question:***<br>Is maternal medication consistent?<br>*Warning shown if field's value is equal to no: '"no" can not be combined with another option, please correct'*	

6. TANGO-DM - Family history

Number	Question	Answers
	This page will only show details if all criteria are ok	

6.1	***If 'Is randomization allowed' is equal to 'yes' answer this question:***<br>Family history of diabetes mellitus (1st or 2nd degree)	◯ yes<br>◯ no<br>◯ unknown
6.2	***If 'Is randomization allowed' is equal to 'yes' answer this question:***<br>Family history of gestational diabetes mellitus (1st or 2nd degree)	◯ yes<br>◯ no<br>◯ unknown
6.3	***If 'Is randomization allowed' is equal to 'yes' answer this question:***<br>Family history of hypertension (1st or 2nd degree)	◯ yes<br>◯ no<br>◯ unknown
6.4	***If 'Is randomization allowed' is equal to 'yes' answer this question:***<br>Family history of preeclampsia (1st or 2nd degree)	◯ yes<br>◯ no<br>◯ unknown
6.5	***If 'Is randomization allowed' is equal to 'yes' answer this question:***<br>Family history of congenital defects (1st or 2nd degree)	◯ yes<br>◯ no<br>◯ unknown

7. TANGO-DM - Obstetric history

Number	Question	Answers
	This page will only show details if all criteria are ok	
7.1	***If 'Is randomization allowed' is equal to 'yes' answer this question:***<br>Gravidity<br>*Notice shown if field's value is larger than 10: 'Gravidity entered is above 10. This is unusual, is this correct?'*<br>*Warning shown if field's value is smaller than 1: 'Gravidity should be at least 1, please correct'*<br>*Warning shown if field's value is larger than or equal to 20: 'Gravidity above 20 must be an error, please correct'*	
7.2	***If 'Is randomization allowed' is equal to 'yes' answer this question:***<br>Parity<br>*Notice shown if field's value is larger than 10: 'Parity entered is above 10. This is unusual, is this correct?'*<br>*Warning shown if field's value is smaller than 0: 'Parity mus be an positive number or 0'*<br>*Warning shown if field's value is larger than or equal to 20: 'A parity above 20 must be an error, please correct'*	

7.2.1 ***If 'Parity' is not equal to '""' answer this question:***
Is Gravidity more than or equal to Parity?
Warning shown if field's value is equal to no: 'Parity entered is greater than gravidity. Please check both gravidity and parity.'

7.2.1.1 ***If 'Is Gravidity more than or equal to Parity?' is equal to 'no' answer this question:***
Is Gravidity more than or equal to Parity?
Warning shown if field's value is equal to no: 'Parity entered is greater than gravidity. Please check both gravidity and parity.'

7.2.2 ***If 'Parity' is bigger than '0' answer this question:***
Live births
Notice shown if field's value is larger than 10: 'Living children entered is above 10. This is unusual, is this correct?'
Warning shown if field's value is smaller than 0: 'This must be a positive number, please correct'
Warning shown if field's value is larger than or equal to 20: '20 or more must be an error, please correct'

7.1.1 ***If 'Gravidity' is bigger than '1' answer this question:***
Miscarriage - spontanious abortion
Notice shown if field's value is larger than 10: 'Miscarriages entered is above 10. This is unusual, is this correct?'
Warning shown if field's value is smaller than 0: 'This must be a positive number or 0, please correct'
Warning shown if field's value is larger than or equal to 20: '20 or above must be an error, please correct'

7.1.2 ***If 'Gravidity' is bigger than '1' answer this question:***
APLA
Notice shown if field's value is larger than 10: 'APLA entered is above 10. This is unusual, is this correct?'
Warning shown if field's value is smaller than 0: 'This must be a positive number, please correct'
Warning shown if field's value is larger than or equal to 20: '20 or more must be an error, please correct'

7.1.3 ***If 'Gravidity' is bigger than '1' answer this question:***
EUG
Notice shown if field's value is larger than 10: 'EUG entered is above 10. This is unusual, is this correct?'
Warning shown if field's value is smaller than 0: 'This must be a positive number, please correct'
Warning shown if field's value is larger than or equal to 20: '20 or more must be an error, please correct'

7.2.3 ***If 'Parity' is bigger than '0' answer this question:***
Intra-uterine death (>16 weeks)
Notice shown if field's value is larger than 10: 'Intra-uterine death entered is above 10. This is unusual, is this correct?'
Warning shown if field's value is smaller than 0: 'This must be a positive number or 0'
Warning shown if field's value is larger than or equal to 20: '20 or above must be an error, please correct'

7.2.4 ***If 'Parity' is bigger than '0' answer this question:***
Is parity (the number of ongoing pregnancies over 16 weeks) > 0?
- yes
- no

7.2.4.1	***If 'Is parity (the number of ongoing pregnancies over 16 weeks) > 0?' is equal to 'yes' answer this question:***<br>Any previous pregnancy with gestational diabetes?	○ no (no GDM in any previous pregnancies)<br>○ yes<br>○ unknown
7.2.4.1.1	***If 'Any previous pregnancy with gestational diabetes?' is equal to 'yes' answer this question:***<br>How many pregnancies with gestational diabetes?	○ 1<br>○ 2<br>○ >2<br>○ unknown
7.2.4.1.2	***If 'Any previous pregnancy with gestational diabetes?' is equal to 'yes' answer this question:***<br>Any pregnancy with GDM treated with insulin?	○ yes<br>○ no<br>○ unknown
7.2.4.2	***If 'Is parity (the number of ongoing pregnancies over 16 weeks) > 0?' is equal to 'yes' answer this question:***<br>Any previous pregnancy with pregnancy induced hypertension (PIH)	○ no (no PIH in any previous pregnancies)<br>○ yes<br>○ unknown
7.2.4.3	***If 'Is parity (the number of ongoing pregnancies over 16 weeks) > 0?' is equal to 'yes' answer this question:***<br>Any previous pregnancy with preeclampsia (PE)?	○ no (no PE in any previous pregnancies)<br>○ yes<br>○ unknown
7.2.4.4	***If 'Is parity (the number of ongoing pregnancies over 16 weeks) > 0?' is equal to 'yes' answer this question:***<br>Any previous pregnancies with HELLP syndrome?	○ no (no HELLP in any previous pregnancies)<br>○ yes<br>○ unknown
7.2.4.5	***If 'Is parity (the number of ongoing pregnancies over 16 weeks) > 0?' is equal to 'yes' answer this question:***<br>Any previous pregnancy with a preterm delivery (<37 weeks)?	○ no (no preterm delivery in any previous pregnancies)<br>○ yes<br>○ unknown
7.2.4.6	***If 'Is parity (the number of ongoing pregnancies over 16 weeks) > 0?' is equal to 'yes' answer this question:***<br>A caesarean section (primary or secondary) in the past?	○ no (no caesarean section in any previous pregnancies)<br>○ yes<br>○ unknown
7.2.4.7	***If 'Is parity (the number of ongoing pregnancies over 16 weeks) > 0?' is equal to 'yes' answer this question:***<br>Any HPP (hemorrhagia postpartum, fluxus (blood loss >1L) in the past?	○ no (no HPP (fluxus) in the past)<br>○ yes<br>○ unknown
7.2.4.8	***If 'Is parity (the number of ongoing pregnancies over 16 weeks) > 0?' is equal to 'yes' answer this question:***<br>Please complete the following questions for all previous pregnancies > 16 weeks	

8. TANGO-DM - Current pregnancy

Number	Question	Answers
	This page will only show details if all criteria are ok	
8.1	***If 'Is randomization allowed' is equal to 'yes' answer this question:***<br>Mode of conception	○ spontaneous<br>○ Clomifene ovulation induction<br>○ IUI<br>○ IVF/ICSI<br>○ egg cell donation<br>○ unknown
8.2	***If 'Is randomization allowed' is equal to 'yes' answer this question:***<br>Height<br>*Warning shown if field's value is smaller than 140: 'This should be 140 or above'*<br>*Warning shown if field's value is larger than 220: 'This should be 220 or less'*	______ cm
8.3	***If 'Is randomization allowed' is equal to 'yes' answer this question:***<br>Weight at start of pregnancy<br>*Warning shown if field's value is smaller than 40: 'This should be 40 or above'*<br>*Warning shown if field's value is larger than 200: 'This should be 200 or less'*	______ kg
8.4	***If 'Is randomization allowed' is equal to 'yes' answer this question:***<br>Maternal weight at time of delivery / last predelivery appointment<br>*Warning shown if field's value is smaller than 40: 'This should be 40 or above'*<br>*Warning shown if field's value is larger than 200: 'This should be 200 or less'*	______ kg
8.5	***If 'Is randomization allowed' is equal to 'yes' answer this question:***<br>Blood pressure systolic (intake)<br>*Warning shown if field's value is smaller than 50: 'This should be 50 or above'*<br>*Warning shown if field's value is larger than 250: 'This should be 250 or less'*	______ mmHg
8.6	***If 'Is randomization allowed' is equal to 'yes' answer this question:***<br>Blood pressure diastolic (intake)<br>*Warning shown if field's value is smaller than 30: 'This should be 30 or above'*<br>*Warning shown if field's value is larger than 200: 'This should be 200 or less'*	______ mmHg

8.7	***If 'Is randomization allowed' is equal to 'yes' answer this question:***<br>Smoking during pregnancy	◯ no<br>◯ quit in first trimester<br>◯ quit later in pregnancy<br>◯ yes<br>◯ unknown
8.8	***If 'Is randomization allowed' is equal to 'yes' answer this question:***<br>Alcohol during pregnancy	◯ yes<br>◯ no<br>◯ unknown
8.9	***If 'Is randomization allowed' is equal to 'yes' answer this question:***<br>Glucose value in first trimester	◯ yes, a fasting glucose was done<br>◯ yes, a random glucose was done<br>◯ yes, other<br>◯ no
8.9.1	***If 'Glucose value in first trimester' is not equal to 'no' answer this question:***<br>Glucose value was<br>*Warning shown if field's value is smaller than 1: 'this should be 1.0 or above'*<br>*Warning shown if field's value is larger than 25.0: 'This should be 25.0 or below'*	[ ] mmol/l
8.10	***If 'Is randomization allowed' is equal to 'yes' answer this question:***<br>Did patient receive routine check ups from midwifes at midwifery practice or midwifes/gynecologist at hospital?	◯ midwives (midwivery practice)<br>◯ midwives/gynecologist (hospital)<br>◯ both
8.11	***If 'Is randomization allowed' is equal to 'yes' answer this question:***<br>What is the total number of ultrasounds performed in this pregnancy (including SEO/GUO etc.)?<br>*Warning shown if field's value is smaller than 1: 'A value between 1 and 20 is expected'*<br>*Warning shown if field's value is larger than 20: 'A value between 1 and 20 is expected'*	[ ]
8.12	***If 'Is randomization allowed' is equal to 'yes' answer this question:***<br>Was there a need for CTG monitoring in pregnancy?	◯ yes<br>◯ no<br>◯ unknown
8.13	***If 'Is randomization allowed' is equal to 'yes' answer this question:***<br>Did the patient see a dietitian?	◯ yes<br>◯ no<br>◯ unknown
8.14	***If 'Is randomization allowed' is equal to 'yes' answer this question:***<br>Did the patient see a endocrinologist or internal medicine specialist?	◯ yes<br>◯ no<br>◯ unknown

8.15	***If 'Is randomization allowed' is equal to 'yes' answer this question:***<br>Did the patient see a physician assistent (diabetes verpleegkundige)	◯ yes<br>◯ no<br>◯ unknown
8.16	***If 'Randomization completed' is equal to 'yes' answer this question:***<br>Did the patient initially receive the treatment of randomization?	◯ yes<br>◯ no
8.17	***If 'Is randomization allowed' is equal to 'yes' answer this question:***<br>Did the patient keep daily glucose curves?	◯ yes<br>◯ no
8.18	***If 'Is randomization allowed' is equal to 'yes' answer this question:***<br>Was GDM under control with dietary restrictions?	◯ yes<br>◯ no<br>◯ unknown
8.18.1	***If 'Was GDM under control with dietary restrictions?' is equal to 'no' answer this question:***<br>Was there a need for medication to control GDM?	◯ yes<br>◯ no<br>◯ unknown
8.18.1.1	***If 'Was there a need for medication to control GDM?' is equal to 'yes' answer this question:***<br>When in pregnancy did the patient start with medication?<br>*Warning shown if field's value is smaller than 16: 'A number between 16 and 43 is expected'*<br>*Warning shown if field's value is larger than 43: 'A number between 16 and 43 is expected'*	______ weeks
8.18.1.2	***If 'Was there a need for medication to control GDM?' is equal to 'yes' answer this question:***<br>-<br>*Warning shown if field's value is smaller than 0: 'A number between 0 and 6 is expected'*<br>*Warning shown if field's value is larger than 6: 'A number between 0 and 6 is expected'*	______ days
8.18.1.3	***If 'Was there a need for medication to control GDM?' is equal to 'yes' answer this question:***<br>Medication used during the study	☐ metformin only<br>☐ metformin and glibenclamide<br>☐ glibenclamide only<br>☐ therapy with metformin and insulin<br>☐ therapy with insulin only
8.19	***If 'Randomized treatment' is equal to '1' answer this question:***<br>Was there a need for a repeat OGTT?	◯ yes<br>◯ no<br>◯ unknown
8.19.1	***If 'Was there a need for a repeat OGTT?' is equal to 'yes' answer this question:***<br>What were the abnormal values of repeat OGTT?	☐ there were no abnormal values<br>☐ fasting glucose > 7.1<br>☐ 2-hour OGTT > 8.5

8.19.2	***If 'Was there a need for a repeat OGTT?' is equal to 'yes' answer this question:***<br>What was the reason for repeat OGTT?	☐ suspected macrosomia/estimated fetal weight >p90<br>☐ polyhydramnion<br>☐ clinical signs of GDM<br>☐ other
8.19.1.1	***If 'What were the abnormal values of repeat OGTT?' is not equal to 'there were no abnormal values' answer this question:***<br>If repeat OGTT was abnormal, did the patient receive treatment?	○ yes dietary restrictions and glucose monitoring only<br>○ yes medication<br>○ no<br>○ unknown

9. TANGO-DM - Pregnancy complications

Number	Question	Answers
	This page will only show details if all criteria are ok	
	Please select whether the following complications occured during the current pregnancy	
	Pregnancy complications - maternal	
9.1	***If 'Is randomization allowed' is equal to 'yes' answer this question:***<br>Pregnancy induced hypertension	○ no<br>○ yes (without medication)<br>○ yes (with medication, for instance labetolol or aldomet)<br>○ yes (unknown whether medication was used)<br>○ unknown
9.2	***If 'Is randomization allowed' is equal to 'yes' answer this question:***<br>Preeclampsia (hypertension with albuminuria)	○ yes<br>○ no<br>○ unknown
9.3	***If 'Is randomization allowed' is equal to 'yes' answer this question:***<br>HELLP	○ yes<br>○ no<br>○ unknown
9.4	***If 'Is randomization allowed' is equal to 'yes' answer this question:***<br>Trombo-embolic complications (deep venous trombosis or lung embolus)	○ yes<br>○ no<br>○ unknown
	Pregnancy complications - fetal	

9.5	***If 'Is randomization allowed' is equal to 'yes' answer this question:***<br>Structural defects	○ no<br>○ yes
9.5.1	***If 'Structural defects' is equal to 'yes' answer this question:***<br>Structural defects	☐ yes (central nervous system, including spina bifida and anencephaly)<br>☐ yes skeletal system (including caudal regression syndrome, limb defects and sacral agenesis)<br>☐ yes (cardiovascular,yes (cardiovascular, including transposition of the great vessels, septal defects, single umbilical artery (SUA), coarctation of the aorta)<br>☐ yes (gastrointestinal, including duodenal atresia)<br>☐ yes but unknown which system<br>☐ other
9.6	***If 'Is randomization allowed' is equal to 'yes' answer this question:***<br>Any abnormalities found on ultrasound in pregnancy	○ yes<br>○ no<br>○ unknown
9.7	***If 'Is randomization allowed' is equal to 'yes' answer this question:***<br>Please select all applicable abnormalities	☐ macrosomia (EFW>p90 or FAC >p90 or mentioned in conclusion)<br>☐ intra-uterine growth restriction (IUGR, EFW< p10 or FAC< p10 or mentioned in conclusion)<br>☐ polyhydramnios<br>☐ oligohydramnios
9.8	***If 'Is randomization allowed' is equal to 'yes' answer this question:***<br>Corticosteroids used? (for instance because of imminent premature birth/dreigende vroeggeboorte)	○ yes<br>○ no<br>○ unknown
9.9	***If 'Is randomization allowed' is equal to 'yes' answer this question:***<br>Intrauterine death	○ yes<br>○ no<br>○ unknown
9.9.1	***If 'Intrauterine death' is equal to 'yes' answer this question:***<br>Date of intrauterine death<br>*Warning shown if field's value is larger than NOW: 'This date is in the future, please check'*	[ ] [ ] [ ] *(dd-mm-yyyy)*

9.9.1.1	***If 'Date of intrauterine death' is not equal to '""' answer this question:***<br>Is date of IUVD between estimated date of delivery – 168 days (GA 16 weeks) and estimated date of delivery + 21 days (GA 43 weeks)?<br>*Warning shown if field's value is equal to no: 'Date of IUVD entered does not correspond with expected value based on estimated date of delivery (gestational age 43 weeks). Please check whether date of IUVD and estimated date of delivery are correct.'*	
9.9.1.1.1	***If 'Is date of IUVD between estimated date of delivery – 168 days (GA 16 weeks) and estimated date of delivery + 21 days (GA 43 weeks)?' is equal to 'no' answer this question:***<br>Is date of IUVD between estimated date of delivery – 168 days (GA 16 weeks) and estimated date of delivery + 21 days (GA 43 weeks)?<br>*Warning shown if field's value is equal to no: 'Date of IUVD entered does not correspond with expected value based on estimated date of delivery (gestational age 43 weeks). Please check whether date of IUVD and estimated date of delivery are correct.'*	
9.10	***If 'Is randomization allowed' is equal to 'yes' answer this question:***<br>Was patient admitted to the hospital during pregnancy other than delivery?	○ yes<br>○ no<br>○ unknown
9.10.1	***If 'Was patient admitted to the hospital during pregnancy other than delivery?' is equal to 'yes' answer this question:***<br>Admission	

10. TANGO-DM - Delivery

Number	Question	Answers
	This page will only show details if all criteria are ok	
	Delivery guidance	
10.1	***If 'Is randomization allowed' is equal to 'yes' answer this question:***<br>Was the delivery a home birth or hospital birth	○ home birth<br>○ hospital birth guided by primary care midwife (poliklinische partus)<br>○ labour guided by primary care midwife (home or hospital) but admitted to hospital care givers during labour<br>○ hospital birth guided by hospital care givers

10.1.1	***If 'Was the delivery a home birth or hospital birth' is equal to 'labour guided by primary care midwife (home or hospital) but admitted to hospital care givers during labour' answer this question:***<br>What was the reason care was transferred from primary care to hospital care givers?	☐ pain relief<br>☐ ailure to progress during dilation (stage 1)<br>☐ failure to progress in pushing phase<br>☐ fetal distress<br>☐ meconium in amniotic fluid<br>☐ postpartum
	Delivery first stage	
10.2	***If 'Is randomization allowed' is equal to 'yes' answer this question:***<br>Onset of labour	○ spontaneously<br>○ primary Caesarean section<br>○ induction
	Delivery first stage - induction	
10.2.2	***If 'Onset of labour' is equal to 'induction' answer this question:***<br>Reason for induction	☐ elective<br>☐ GDM<br>☐ ruptured membranes<br>☐ hypertension<br>☐ preeclampsia<br>☐ HELLP syndrome<br>☐ maternal: blood glucose dysregulation<br>☐ maternal: other<br>☐ fetal: suspected macrosomia<br>☐ fetal: IUGR<br>☐ fetal: no movements<br>☐ fetal: heart rate anomaly<br>☐ fetal: oligohydramnios<br>☐ fetal: meconium<br>☐ fetal: other<br>☐ unknown
10.2.3	***If 'Onset of labour' is equal to 'induction' answer this question:***<br>Method of induction	☐ foley catheter / mechanical<br>☐ prostaglandins<br>☐ amniotomy<br>☐ oxytocin<br>☐ other<br>☐ unknown
	Delivery first stage - primary Caesarean	

10.2.5	***If 'Onset of labour' is equal to 'primary Caesarean section' answer this question:***<br>Indication for primary Caesarean	☐ elective: breech<br>☐ elective: obstetric history (previous Caesarean section)<br>☐ elective: obstetric history (total sphincter rupture)<br>☐ elective: obstetric history (other)<br>☐ fetal distress<br>☐ fetal: IUGR<br>☐ fetal: other<br>☐ maternal: hypertension<br>☐ maternal: preeclampsia<br>☐ maternal: HELLP syndrome<br>☐ maternal: other<br>☐ unknown
	Delivery second stage	
10.2.7	***If 'Onset of labour' is not equal to 'primary Caesarean section' answer this question:***<br>Pain relief during delivery	○ no<br>○ yes
10.2.7.1	***If 'Pain relief during delivery' is equal to 'yes' answer this question:***<br>Pain relief during delivery	☐ opioid subcutanious (pethidine)<br>☐ opioid intravenous (remifentanil)<br>☐ nitrous oxide<br>☐ epidural<br>☐ other<br>☐ unknown
10.2.8	***If 'Onset of labour' is not equal to 'primary Caesarean section' answer this question:***<br>Medication during labour	☐ oxytocin<br>☐ antibiotics<br>☐ tocolytics<br>☐ glucose/insulin intravenous<br>☐ antihypertensive agents intravenous (for instance labetolol)<br>☐ other<br>☐ none<br>☐ unknown
10.2.8.1	***If 'Medication during labour' is equal to 'other' answer this question:***<br>Specify other medication	
10.3	Is medication during labour consistent	
10.3.1	***If 'Is medication during labour consistent' is equal to 'no' answer this question:***<br>Is medication during labour consistent<br>*Warning shown if field's value is equal to no: '"none' should not be combined with other options, please correct'*	

10.2.9	***If 'Onset of labour' is not equal to 'primary Caesarean section' answer this question:***<br>Fever during labour	○ no<br>○ yes (>38.0 °C <38.5 °C)<br>○ yes (> 38.5 °C)<br>○ unknown
10.2.10	***If 'Onset of labour' is not equal to 'primary Caesarean section' answer this question:***<br>Fetal presentation	○ cephalic<br>○ breech<br>○ other
10.2.11	***If 'Onset of labour' is not equal to 'primary Caesarean section' answer this question:***<br>Route of delivery	○ spontaneously<br>○ instrumental: vacuum extraction<br>○ instrumental: forcipal extraction<br>○ secondary Caesarean section
10.4	Show Instrumental question	
10.4.1	***If 'Show Instrumental question' is equal to 'Yes' answer this question:***<br>Indication vacuum / forcipal extraction	☐ fetal distress<br>☐ failure to progress<br>☐ maternal indication<br>☐ other fetal indication<br>☐ unknown
10.2.11.1	***If 'Route of delivery' is equal to 'secondary Caesarean section' answer this question:***<br>Indication secondary Caesarean section	○ fetal distress<br>○ failure to progress<br>○ failed induction<br>○ maternal indication<br>○ failed vacuum / forcipal extraction<br>○ other fetal indication<br>○ unknown
10.2.12	***If 'Onset of labour' is not equal to 'primary Caesarean section' answer this question:***<br>Were maneuvers used because of shoulder dystocia?	☐ no (no shoulder dystocia)<br>☐ traction to fetal head<br>☐ McRoberts<br>☐ Rubin<br>☐ all-fours<br>☐ manual delivery of posterior arm<br>☐ intentional breaking of clavicle<br>☐ shoulderdystocia but unknown which maneuvers were used<br>☐ other
10.5	***If 'Is randomization allowed' is equal to 'yes' answer this question:***<br>Amniotic fluid	○ clear<br>○ meconium<br>○ unknown

10.6	***If 'Is randomization allowed' is equal to 'yes' answer this question:***<br>Ruptured membranes >24 hours (langdurig gebroken vliezen)	○ no<br>○ yes >24 hours<br>○ yes >48 hours<br>○ unknown
10.7	***If 'Is randomization allowed' is equal to 'yes' answer this question:***<br>Delivery of placenta	○ spontaneously/controlled cord traction<br>○ manual removal in operating room<br>○ during Caesarean section<br>○ unknown
10.8	***If 'Is randomization allowed' is equal to 'yes' answer this question:***<br>Total blood loss<br>*Warning shown if field's value is smaller than 1: 'This must be 1 or above'*<br>*Warning shown if field's value is larger than 7000: 'This must be 7000 or less'*	______ ml
10.9	***If 'Is randomization allowed' is equal to 'yes' answer this question:***<br>Blood transfusion	○ yes<br>○ no<br>○ unknown
10.2.13	***If 'Onset of labour' is not equal to 'primary Caesarean section' answer this question:***<br>Perineum	☐ no lacerations<br>☐ first / second degree laceration(s)<br>☐ third degree laceration (totaalruptuur)<br>☐ episiotomy<br>☐ unknown

11. TANGO-DM - Neonatal data

Number	Question	Answers
11.1	Is randomization allowed	
	This page will only show details if all criteria are ok	
11.1.2	***If 'Is randomization allowed' is equal to 'yes' answer this question:***<br>Neonatal date of birth<br>*Warning shown if field's value is larger than NOW: 'This date is in the future, please check'*	___ ___ ___ *(dd-mm-yyyy)*

11.1.2.1	***If 'Neonatal date of birth' is not equal to '""' answer this question:***<br>Is Date of neonatal birth as expected based on estimated date of delivery<br>*Warning shown if field's value is equal to no: 'Date of neonatal date of birth does not correspond with expected value based on estimated date of delivery (gestational age less then 16 or greater than 43 weeks). Please check whether neonatal date of birth and estimated date of delivery are correct.'*	
11.1.2.1.1	***If 'Is Date of neonatal birth as expected based on estimated date of delivery' is equal to 'no' answer this question:***<br>Is Date of neonatal birth as expected based on estimated date of delivery<br>*Warning shown if field's value is equal to no: 'Date of neonatal date of birth does not correspond with expected value based on estimated date of delivery (gestational age less then 16 or greater than 43 weeks). Please check whether neonatal date of birth and estimated date of delivery are correct.'*	
11.1.3	***If 'Is randomization allowed' is equal to 'yes' answer this question:***<br>Gestational age at birth<br>*Warning shown if field's value is smaller than 16: 'This should be 16 or above'*<br>*Warning shown if field's value is larger than 43: 'This should be 43 or less'*	[ ] weeks
11.1.4	***If 'Is randomization allowed' is equal to 'yes' answer this question:***<br>+<br>*Warning shown if field's value is smaller than 0: 'This should be 0 or above'*<br>*Warning shown if field's value is larger than or equal to 7: 'This should be less than 7'*	[ ] days
11.1.5	***If 'Is randomization allowed' is equal to 'yes' answer this question:***<br>Live birth	○ yes<br>○ no (deceased during labour)<br>○ no (deceased before labour)
11.2	Is live birth in line with IUVD in pregnancy complications<br>*Warning shown if field's value is equal to no: 'Live birth can not be no (deceased before labour) if IUVD in section ‘pregnancy complications’ is no or Live birth can not be yes if IUVD. Please change one of these'*	
11.2.1	***If 'Is live birth in line with IUVD in pregnancy complications' is equal to 'no' answer this question:***<br>Is live birth in line with IUVD in pregnancy complications<br>*Warning shown if field's value is equal to no: 'Live birth can not be no (deceased before labour) if IUVD in section ‘pregnancy complications’ is no or Live birth can not be yes if IUVD. Please change one of these'*	

11.1.6	***If 'Is randomization allowed' is equal to 'yes' answer this question:***<br>Neonatal death	◯ no<br>◯ yes (IUVD)<br>◯ yes <24 hours postpartum<br>◯ yes >24 hours postpartum
11.3	Is Neonatal death consistent with other data<br>*Warning shown if field's value is equal to no: 'Data entered is inconsistent with previous entries. Neonatal death less then 24 hours and more then 24 hours postpartum are not allowed if IUVD in section 'Pregnancy complications' is yes and Neonatal live birth is no (deceased before labour)'*	
11.3.1	***If 'Is Neonatal death consistent with other data' is equal to 'no' answer this question:***<br>Is Neonatal death consistent with other data<br>*Warning shown if field's value is equal to no: 'Data entered is inconsistent with previous entries. Neonatal death less than 24 hours and more than 24 hours postpartum are not allowed if IUVD in section 'Pregnancy complications' is yes and Neonatal live birth is no (deceased before labour)'*	
11.1.7	***If 'Is randomization allowed' is equal to 'yes' answer this question:***<br>ShowDateOfDeath	
11.1.7.1	***If 'ShowDateOfDeath' is equal to 'yes' answer this question:***<br>Date of death<br>*Warning shown if field's value is larger than NOW: 'Date is in the future, please check'*	[ ] [ ] [ ] *(dd-mm-yyyy)*
11.1.7.1.1	***If 'Date of death' is not equal to """ answer this question:***<br>Is the date of death in case of deceased before labour identical to date of IUVD in section 'Pregnancy complications'<br>*Warning shown if field's value is equal to no: 'Date of death when deceased before labour has to be identical to date of intrauterine death in the form Pregnancy complications'*	
11.1.7.1.1.1	***If 'Is the date of death in case of deceased before labour identical to date of IUVD in section 'Pregnancy complications'' is equal to 'no' answer this question:***<br>Is the date of death in case of deceased before labour identical to date of IUVD in section 'Pregnancy complications'<br>*Warning shown if field's value is equal to no: 'Date of death when deceased before labour has to be identical to date of intrauterine death in the form Pregnancy complications'*	

11.1.7.1.2	***If 'Date of death' is not equal to """ answer this question:***<br>Is Neonatal date of death postpartum after neonatal date of birth.<br>*Warning shown if field's value is equal to no: 'Date entered is inconsistent with previous entry: date of death postpartum can not be before neonatal date of birth'*	
11.1.7.1.2.1	***If 'Is Neonatal date of death postpartum after neonatal date of birth.' is equal to 'no' answer this question:***<br>Is Neonatal date of death postpartum after neonatal date of birth.<br>*Warning shown if field's value is equal to no: 'Date entered is inconsistent with previous entry: date of death postpartum can not be before neonatal date of birth'*	
11.1.8	***If 'Is randomization allowed' is equal to 'yes' answer this question:***<br>Gender	○ female<br>○ male<br>○ unknown
11.1.9	***If 'Is randomization allowed' is equal to 'yes' answer this question:***<br>Apgar score 1 minute postpartum<br>*Warning shown if field's value is smaller than 0: 'This should 0 or above'*<br>*Warning shown if field's value is larger than 10: 'This should be 10 or less'*	
11.1.10	***If 'Is randomization allowed' is equal to 'yes' answer this question:***<br>Apgar score 5 minutes postpartum<br>*Warning shown if field's value is smaller than 0: 'This should be 0 or more'*<br>*Warning shown if field's value is larger than 10: 'This should be 10 or less'*	
11.1.11	***If 'Is randomization allowed' is equal to 'yes' answer this question:***<br>Apgar score 10 minutes postpartum (if available)<br>*Warning shown if field's value is smaller than 0: 'This should be 0 or more'*<br>*Warning shown if field's value is larger than 10: 'This should be 10 or less'*	
11.1.12	***If 'Is randomization allowed' is equal to 'yes' answer this question:***<br>Birth weight<br>*Warning shown if field's value is smaller than 100: 'This should be 100 or more'*<br>*Warning shown if field's value is larger than 7000: 'This should be 7000 or less'*	grams

11.1.13	***If 'Is randomization allowed' is equal to 'yes' answer this question:***<br>Fracture	◯ none<br>◯ humerus<br>◯ clavicle<br>◯ other<br>◯ unknown
11.1.14	***If 'Is randomization allowed' is equal to 'yes' answer this question:***<br>Erbs palsy (Erbse parese)	◯ no<br>◯ yes<br>◯ unknown
11.1.15	***If 'Is randomization allowed' is equal to 'yes' answer this question:***<br>Was neonatal glucose testing done?	◯ yes<br>◯ no<br>◯ unknown
11.1.15.1	***If 'Was neonatal glucose testing done?' is equal to 'yes' answer this question:***<br>Any neonatal glucose value between 2.0-2.6mmol/l (2.0 tm 2.6) during admission in hospital?	◯ no<br>◯ yes 1 value between 2.0 and 2.6<br>◯ yes more than 1 value between 2.0 and 2.6<br>◯ unknown
11.1.15.2	***If 'Was neonatal glucose testing done?' is equal to 'yes' answer this question:***<br>Any neonatal glucose value <2.0mmol/l during admission in hospital?	◯ no<br>◯ yes 1 value <2.0<br>◯ yes more than 1 value <2.0<br>◯ unknown
11.1.15.3	***If 'Was neonatal glucose testing done?' is equal to 'yes' answer this question:***<br>Screenshot send?	◯ yes<br>◯ no
11.1.15.3.1	***If 'Screenshot send?' is equal to 'yes' answer this question:***<br>First neonatal glucose postpartum<br>*Warning shown if field's value is smaller than 0.0: 'This should be 0.0 or mre'*<br>*Warning shown if field's value is larger than 25.0: 'This should be 25.0 or less'*	________ mmol/L
11.1.15.3.2	***If 'Screenshot send?' is equal to 'yes' answer this question:***<br>Date of first neonatal glucose testing postpartum	___ ___ ___ *(dd-mm-yyyy)*
11.4	Is date in line with Neonatal date of birth?<br>*Warning shown if field's value is equal to no: 'Date entered is before date of birth or too long after date of birth.'*	
11.4.1	***If 'Is date in line with Neonatal date of birth?' is equal to 'no' answer this question:***<br>Is date in line with Neonatal date of birth?<br>*Warning shown if field's value is equal to no: 'Date entered is before date of birth or too long after date of birth.'*	

11.1.15.3.3	***If 'Screenshot send?' is equal to 'yes' answer this question:***<br>Time of first neonatal glucose testing postpartum	___ : ___ *(hh:mm)*
11.1.15.3.4	***If 'Screenshot send?' is equal to 'yes' answer this question:***<br>Second neonatal glucose value postartum<br>*Warning shown if field's value is smaller than 0.0: 'This should be 0.0 or more'*<br>*Warning shown if field's value is larger than 25.0: 'This should be 25.0 or less'*	___ mmol/L
11.1.15.3.5	***If 'Screenshot send?' is equal to 'yes' answer this question:***<br>Date of second neonatal glucose testing postpartum	___ ___ ___ *(dd-mm-yyyy)*
11.5	Is second date in line with date of previous glucose date<br>*Warning shown if field's value is equal to no: 'Date entered is before the date of previous glucose value.'*	
11.5.1	***If 'Is second date in line with date of previous glucose date' is equal to 'no' answer this question:***<br>Is second date in line with date of previous glucose date<br>*Warning shown if field's value is equal to no: 'Date entered is before the date of previous glucose value or not withing 5 days of date of birth'*	
11.1.15.3.6	***If 'Screenshot send?' is equal to 'yes' answer this question:***<br>Time of second neonatal glucose testing postpartum	___ : ___ *(hh:mm)*
11.1.15.3.7	***If 'Screenshot send?' is equal to 'yes' answer this question:***<br>Third neonatal glucose value postartum<br>*Warning shown if field's value is smaller than 0.0: 'This should be 0.0 or more'*<br>*Warning shown if field's value is larger than 25.0: 'This should be 25.0 or less'*	___ mmol/L
11.1.15.3.8	***If 'Screenshot send?' is equal to 'yes' answer this question:***<br>Date of third neonatal glucose testing postpartum	___ ___ ___ *(dd-mm-yyyy)*
11.6	Is third date in line with date of previous glucose date<br>*Warning shown if field's value is equal to no: 'Date entered is before the date of previous glucose value.'*	
11.6.1	***If 'Is third date in line with date of previous glucose date' is equal to 'no' answer this question:***<br>Is third date in line with date of previous glucose date<br>*Warning shown if field's value is equal to no: 'Date entered is before the date of previous glucose value or not withing 5 days of date of birth.'*	
11.1.15.3.9	***If 'Screenshot send?' is equal to 'yes' answer this question:***<br>Time of third neonatal glucose testing postpartum	___ : ___ *(hh:mm)*

11.1.15.3.10	***If 'Screenshot send?' is equal to 'yes' answer this question:***<br>Fourth neonatal glucose value postartum<br>*Warning shown if field's value is smaller than 0.0: 'This should be 0.0 or more'*<br>*Warning shown if field's value is larger than 25.0: 'This should be 25.0 or less'*	mmol/L
11.1.15.3.11	***If 'Screenshot send?' is equal to 'yes' answer this question:***<br>Date of fourth neonatal glucose testing postpartum	*(dd-mm-yyyy)*
11.1.15.3.11.1	***If 'Date of fourth neonatal glucose testing postpartum' is not equal to '""' answer this question:***<br>Is fourth date in line with date of previous glucose date<br>*Warning shown if field's value is equal to no: 'Date entered is before the date of previous glucose value.'*	
11.1.15.3.11.1.1	***If 'Is fourth date in line with date of previous glucose date' is equal to 'no' answer this question:***<br>Is fourth date in line with date of previous glucose date<br>*Warning shown if field's value is equal to no: 'Date entered is before the date of previous glucose value.'*	
11.1.15.3.12	***If 'Screenshot send?' is equal to 'yes' answer this question:***<br>Time of fourth neonatal glucose testing postpartum	: *(hh:mm)*
11.1.15.3.13	***If 'Screenshot send?' is equal to 'yes' answer this question:***<br>Fifth neonatal glucose value postartum<br>*Warning shown if field's value is smaller than 0.0: 'This should be 0.0 or more'*<br>*Warning shown if field's value is larger than 25.0: 'This should be 25.0 or less'*	mmol/L
11.1.15.3.14	***If 'Screenshot send?' is equal to 'yes' answer this question:***<br>Date of fifth neonatal glucose testing postpartum	*(dd-mm-yyyy)*
11.1.15.3.14.1	***If 'Date of fifth neonatal glucose testing postpartum' is not equal to '""' answer this question:***<br>Is fifth date in line with date of previous glucose date<br>*Warning shown if field's value is equal to no: 'Date entered is before the date of previous glucose value.'*	
11.1.15.3.14.1.1	***If 'Is fifth date in line with date of previous glucose date' is equal to 'no' answer this question:***<br>Is fifth date in line with date of previous glucose date<br>*Warning shown if field's value is equal to no: 'Date entered is before the date of previous glucose value or not withing 5 days of date of birth.'*	
11.1.15.3.15	***If 'Screenshot send?' is equal to 'yes' answer this question:***<br>Time of fifth neonatal glucose testing postpartum	: *(hh:mm)*

11.1.15.3.16	***If 'Screenshot send?' is equal to 'yes' answer this question:***<br>Sixth neonatal glucose value postartum<br>*Warning shown if field's value is smaller than 0.0: 'This should be 0.0 or more'*<br>*Warning shown if field's value is larger than 25.0: 'This should be 25.0 or less'*	[ ] mmol/L
11.1.15.3.17	***If 'Screenshot send?' is equal to 'yes' answer this question:***<br>Date of sixth neonatal glucose testing postpartum	[ ] [ ] [ ] *(dd-mm-yyyy)*
11.7	Is date in line with date of previous glucose date<br>*Warning shown if field's value is equal to no: 'Date entered is before the date of previous glucose value.'*	
11.7.1	***If 'Is date in line with date of previous glucose date' is equal to 'no' answer this question:***<br>Is date in line with date of previous glucose date<br>*Warning shown if field's value is equal to no: 'Date entered is before the date of previous glucose value or not withing 5 days of date of birth.'*	
11.1.15.3.18	***If 'Screenshot send?' is equal to 'yes' answer this question:***<br>Time of sixth neonatal glucose testing postpartum	[ ] : [ ] *(hh:mm)*

12. TANGO-DM - PostPartum

Number	Question	Answers
12.1	Is randomization allowed	
	This page will only show details if all criteria are ok	
12.1.2	***If 'Is randomization allowed' is equal to 'yes' answer this question:***<br>Were mother or child admitted directly postpartum? (including postpartum observation of mother/child)	○ no (mother and child went home directly after delivery)<br>○ yes (maternal admission only)<br>○ yes (maternal and neonatal admission)<br>○ yes (neonatal admission only)
12.1.3	***If 'Is randomization allowed' is equal to 'yes' answer this question:***<br>Show Maternal	
12.1.3.1	***If 'Show Maternal' is equal to 'Yes' answer this question:***<br>Maternal admission	

12.1.4	***If 'Is randomization allowed' is equal to 'yes' answer this question:***<br>Show Neonatal	
12.1.4.1	***If 'Show Neonatal' is equal to 'Yes' answer this question:***<br>Neonatal Admission	

13. TANGO-DM - End of study

Number	Question	Answers
13.1	Is randomization allowed	
13.1.1	***If 'Is randomization allowed' is equal to 'yes' answer this question:***<br>Was there a protocol violation?	yes<br>no<br>unknown
13.1.1.1	***If 'Was there a protocol violation?' is equal to 'yes' answer this question:***<br>What did the protocol violation entail?	
13.1.2	***If 'Is randomization allowed' is equal to 'yes' answer this question:***<br>Did a Serious Adverse Event (SAE) occur during the study until 6 weeks postpartum? If yes, please report the SAE to the sponsor	yes<br>no<br>unknown
13.2	Please specify if the subject completed the entire course of the study as specified in the protocol or discontinued the study:	completed<br>discontinued
13.2.1	***If 'Please specify if the subject completed the entire course of the study as specified in the protocol or discontinued the study:' is equal to 'discontinued' answer this question:***<br>If discontinued, please specify the most appropriate reason for early termination	subject violates one or more of the inclusion/exclusion criteria<br>adverse event<br>subject died<br>subject lost to follow up<br>subject withdrew consent to use personal data<br>investigator's and/or physician's decision<br>total study is early terminated<br>other reason

13.2.1.1	***If 'If discontinued, please specify the most appropriate reason for early termination' is equal to 'adverse event' answer this question:***<br>Please specify adverse event	
13.2.1.2	***If 'If discontinued, please specify the most appropriate reason for early termination' is equal to 'subject died' answer this question:***<br>Please specify date of death	*(dd-mm-yyyy)*
13.2.1.3	***If 'If discontinued, please specify the most appropriate reason for early termination' is equal to 'subject died' answer this question:***<br>Please specify primary cause of death	
13.2.1.4	***If 'If discontinued, please specify the most appropriate reason for early termination' is equal to 'subject lost to follow up' answer this question:***<br>Please specify date of last contact with subject (visit or call) in this study	*(dd-mm-yyyy)*
13.2.1.5	***If 'If discontinued, please specify the most appropriate reason for early termination' is equal to 'investigator's and/or physician's decision' answer this question:***<br>Please specify date of decision	*(dd-mm-yyyy)*
13.1.3	***If 'Is randomization allowed' is equal to 'yes' answer this question:***<br>Have the subject signed informed consent for follow-up?	○ yes<br>○ no
13.1.3.1	***If 'Have the subject signed informed consent for follow-up?' is equal to 'yes' answer this question:***<br>Have the subject provided contact information to allow follow-up?	○ yes<br>○ no
